# An Indian Woman's Tribute to Lady Dufferin

**Published:** 1888-08-18

**Authors:** 


### AN INDIAN WOMAN'S TRIBUTE TO LADY DUFFERIN.

The most graceful tribute hitherto paid to Lady Dufferin's noble work in India comes from the *Calcutta and M. Gazette*:—

"Our feelings in this matter are shared by thousands and thousands of our sisters throughout the land—and of this we are assured by many signs not likely to come under the observation of the outside world." —Vide *Address of the Women of Utterpara to Lady Dufferin.*

How shall she know the worship we would do her?
The walls are high and she is very far.
How can the women's message reach unto her
Above the tumult of the packed bazaar?
Free Wind of Chait. against the lattice blowing,
Bear thou our thanks lest she depart unknowing.

Go forth across the fields we may not roam in—
Go forth beyond the trees that rim the city—
To whatsoe'er fair place she hath her home in
Who dowered us with wealth of help and pity.
Out of our shadow pass and seek her singing:
"I bear no gifts but Love alone for bringing."

If she have sent her servants in our pain,
If she have fought with Death and dulled his sword,
If she have given back our sick again,
And to our breast the weakling lips restored,
Is it a little thing that she hath wrought?
Then Birth and Death and Motherhood be naught.

Go forth, O Wind, the message on thy wings,
And they shall hear thee pass and bid thee speed,
In reed-roofed hut or white-walled home of kings,
Who have been holpen by her in their need.
All spring shall give thee fragrance, and the wheat
Shall be a golden floorcloth to thy feet.

Haste, for our hearts are with thee—take no rest.
Clear-voiced ambassador from sea to sea,
Proclaim the blessing manifold, confessed,
Of those in darkness, by her hand set free.
Then very softly to her Presence move,
And whisper—"Lady! lo, they know and love!"

